# Mung Bean Peptides Alleviate Dextran-Sulfate-Sodium-Induced Colitis Symptoms in Mice by Protecting the Intestinal Mechanical Barrier and Regulating Gut Microbiota

**DOI:** 10.3390/foods14081363

**Published:** 2025-04-15

**Authors:** Chong Xu, Jingjing Diao, Yuchao Feng, Shu Zhang, Yanan Sheng, Changyuan Wang

**Affiliations:** 1 College of Animal Science and Veterinary Medicine, Heilongjiang Bayi Agricultural University, Daqing 163319, China; byndxuchong@163.com; 2 National Coarse Cereals Engineering Research Center, Daqing 163319, China; 3 College of Food, Heilongjiang Bayi Agricultural University, Daqing 163319, China

**Keywords:** mung bean peptides, DSS, colitis, intestinal barrier, gut microbiota

## Abstract

Ulcerative colitis (UC), an idiopathic and recurrent ailment, substantially influences a patient’s health. Mung bean peptides (MBPs) are bioactive substances derived from mung bean protein that possess notable anti-inflammatory properties. However, their efficacy and underlying mechanisms in UC treatment remain unclear. In this study, the structural characteristics of MBPs were examined by determining various parameters, such as amino acid composition, molecular weight distribution, and peptide sequences, thereby structurally demonstrating their anti-inflammatory potential. The therapeutic effectiveness of MBPs in UC treatment was evaluated by assessing its influence on colon length, histological damage to colonic tissue, and disease activity index of mice suffering from colitis induced by dextran sulfate sodium (DSS). Additionally, the study explored the potential mechanism of action of MBPs in UC by analyzing the intestinal microbiota, inflammatory cytokines in serum, and tight junction (TJ) proteins in the colon tissue of mice. The results revealed that MBPs significantly increased colon length, reduced colonic tissue damage, and decreased the disease activity index in mice with UC. MBPs restored intestinal barrier function by upregulating the expression of ZO-1 and claudin-1 proteins within the colonic tissue of mice with DSS-induced colitis, thereby treating UC. MBPs exerted anti-inflammatory effects by downregulating the amplification of inflammatory cytokines in the serum, improving the gut microbiota structure in mice with colitis, and regulating immune-related signaling pathways. Therefore, there is an experimental basis for the potential use of MBPs as adjunctive therapy in UC.

## 1. Introduction

Ulcerative colitis (UC) is a chronic, non-specific inflammatory condition of the gastrointestinal tract, accompanied by various clinical manifestations such as weight loss, diarrhea, abdominal discomfort, and bloody stools [1]. This disease severely affects human health. The global incidence of UC has significantly increased, imposing substantial burdens on health, society, and the economy [2]. The etiology of UC is complex and may involve multiple factors, including the environment, psychology, epithelial barrier defects, genetic susceptibility, and dysregulated immune responses, resulting in unclear pathogenesis [3]. In recent years, research on UC has increased [4,5,6,7,8]. Based on the possible causes of UC, current medications include corticosteroids, amino salicylates, and antibiotics [9]. However, prolonged use of these drugs can lead to side effects, such as liver problems, fever, hypertension, diabetes, and allergic reactions [10]. Hence, there is an urgent need to identify effective therapies with minimal or no side effects for the prevention and alleviation of UC.

Inflammatory cytokines play a vital role in the development of UC [11]. They are of great significance in regulating inflammation in the mucosal immune systems. Cytokine-targeting therapies are potential therapeutic options for UC [12]. Moreover, intestinal epithelial barrier dysfunction plays a major role in intestinal diseases [13]. The intestinal barrier, which serves as the primary line of defense for the mucosal immune system, physically isolates host immune cells from external stimuli [14]. Evidence suggests that the gut microbiome of patients with UC undergoes significant alterations [15]. There is a close correlation between intestinal barrier function and gut microbiota. Pathogenic intestinal bacteria can disrupt the structural barrier in UC by modifying intestinal tight junction (TJ) proteins, which strengthens the immune system and allows the gut microbiota to migrate, ultimately inducing an inflammatory response [16]. Therefore, maintaining a proper balance of gut microbiota, protecting intestinal mucosal barrier homeostasis, and promoting mucosal healing are considered the main research directions for developing therapeutic drugs for UC [17].

Bioactive peptides refer to various peptides with complex linear and cyclic structures, ranging from dipeptides to longer peptide sequences. These peptides are composed of multiple amino acids (typically between 2 and 20 amino acids) linked by amide bonds in diverse compositions and arrangements. They are more easily digested and absorbed than other types of proteins. These peptides exhibit unique functional properties owing to their specific amino acid compositions, such as antioxidant, anti-fatigue, anti-hypertensive, antibacterial, and immunomodulatory effects [18]. Some studies have utilized the anti-inflammatory effects of peptides to treat UC [19,20]. Mung beans have heat-clearing and detoxifying effects and are high in protein, averaging approximately 22%. Compared to other protein sources, mung bean protein contains a wider variety of amino acids, including leucine, lysine, tyrosine, and valine [21]. The unique amino acid composition and sequence of mung bean protein endow MBPs with specific functions. Recent studies have revealed that MBPs can reverse gut microbial imbalance induced by a high-fat diet, enhance the anti-stress ability of mice, and exert a regulatory effect on immunity [22,23]. In addition, MBPs have been found to exhibit strong anti-inflammatory, antioxidant, hypoglycemic, and hypotensive effects [24,25,26,27]. By activating macrophages, enhancing the viability and phagocytosis of normal macrophages, and inducing the production of cytokines such as interleukins, MBPs exert anti-inflammatory and immunomodulatory effects [28,29]. However, to date, no relevant studies have demonstrated the therapeutic efficacy of MBPs in the treatment of UC. To further investigate whether MBPs can regulate the gut microbiota of mice affected by UC and restore their intestinal barrier function, thus playing an adjuvant therapeutic role in colitis, this study proposed treating mice with MBPs and then inducing UC by administering dextran sulfate sodium (DSS). Subsequently, the phenotypes, cytokine levels in the blood, intestinal wall barrier, and gut microbiota of the mice were measured to examine the effects of MBPs on UC and to analyze their possible mechanisms of action. In conclusion, MBPs can be used as nutritional supplements to safely and effectively prevent the occurrence and development of UC.

## 2. Materials and Methods

### 2.1. MBPs Preparation

Mung bean protein powder (Shandong Zhaoyuan Wenji Food Co., Ltd., Zhaoyuan, Shandong, China) was dissolved in distilled water to prepare a 7% mass sample solution, which was heated to 95 °C and maintained at that temperature for 10 min. Next, the solution temperature was reduced to 55 °C and maintained constant. After adjusting the pH to 7.0 with NaOH solution, 2% papain (by mass fraction) was added to initiate hydrolysis while maintaining the pH at 7.0. After 2.5 h, hydrolysis was terminated by rapidly heating the solution to 95 °C and maintaining it for 10 min to deactivate the enzymes. The solution was then cooled to approximately 25 °C and centrifuged using a TG16-WS centrifugal machine (Shanghai Luxiangyi Centrifuge Instrument Co., Ltd., Shanghai, China) at 5000 rpm for 30 min. The obtained solution was desalted to remove byproducts and impurities generated during peptide synthesis using a multifunctional rolled membrane pilot plant (Xiamen Fumei Technology Co., Ltd., Xiamen, Fujian, China) equipped with an NF1 nanofiltration membrane (Xiamen Fumei Technology Co., Ltd. Xiamen, Fujian, China, 1812 membrane core, 150 Da). The solution was then spray-dried using a spray dryer (YamaTop Technology Trading [Shanghai] Co., Ltd., Shanghai, China, spray pressure of 0.07 MPa; outlet and inlet temperatures of 60 °C and 110 °C respectively) to obtain the MBPs.

### 2.2. Scanning Electron Microscopy

The sample was placed on a conductive adhesive, fixed, gold-coated, and photographed using a Merlin Compact field-emission scanning electron microscope (Carl Zeiss AG, Oberkochen, Baden-Württemberg, German) with an electron beam accelerating voltage of 5 kV, a current of 50 pA, and a probe voltage of 30 kV. The magnification was adjusted to 1000× and 2000× to observe and photograph the microscopic morphologies of the samples.

### 2.3. Fourier Transform Infrared Spectroscopy

A quantity of 0.002 g of the sample and 0.2 g potassium bromide were weighed, mixed thoroughly, and ground evenly before being pressed into tablets. A Nicolet 6700 FTIR (Thermo Electron Co., Madison, WI, USA) was used to scan the spectral bands of the pressed tablets in the range of up to 4,000,400 cm^−1^, with a wave number accuracy of 0.01 cm^−1^, 64 scans, and a resolution of 4 cm^−1^ at room temperature.

### 2.4. Determination of Amino Acid Composition

The sample pretreatment steps were carried out according to the method specified in GB 5009.124-2016 [30]. The test fluid was analyzed using an L-8900 amino acid analyzer (Hitachi Ltd., Tokyo, Japan).

### 2.5. Determination of Relative Molecular Mass Distribution

The MBPs solution was prepared at a concentration of 5 g/L in the mobile phase, filtered through a polyhexanediylhexanediamine membrane with a pore size of 0.22 µm, and analyzed using an Agilent 1260 HPLC (Agilent Technology Co., Ltd., Santa Clara, CA, USA) equipped with an ultraviolet detector. Thyroglobulin bovine (0.5 g/L, relative molecular mass: 670,000), γ-globulins from bovine blood (0.1 g/L, relative molecular mass: 150,000), albumin chicken egg grade VI (0.1 g/L, relative molecular mass: 44,300), and Ribonuclease A type I-A (0.1 g/L, relative molecular mass: 13,700) were used as standard solutions. The chromatographic conditions were as follows: column, TSKGgel G3000SWXL 7.8 mm × 300 mm; mobile phase, 0.1 mol/L Na_2_SO_4_, 0.1 mol/L NaH_2_PO_4_ and Na_2_HPO_4_ buffer solution at pH 6.7; flow rate, 0.5 mL/min; detection wavelengths, 220 nm, 280 nm; column temperature, 30 °C.

### 2.6. Determination of Peptide Sequences

LC-MS/MS was used to analyze the amino acid sequences of the target peptides. Mobile phase A was 0.1% formic acid, and mobile phase B was 0.1% formic acid with 80% acetonitrile. The column was equilibrated with 100% mobile phase A before analysis. The sample was separated using a C18 column (Dr. Maisch GmbH High Performance LC Co., Tübingen, Baden-Württemberg, German, 75 µm × 150 mm, 3 µm) using gradient elution. The flow rate was set to 300 nL/min. The liquid chromatography gradient was as follows: 0–2 min, linear gradient from 2% to 5% B; 2–44 min, linear gradient from 5% to 28% B; 44–51 min, linear gradient from 28% to 40% B; 51–53 min, linear gradient from 40% to 100% B; and 53–60 min, maintained at 100% B. After peptide separation, the peptides were analyzed using a Q-Exactive HF mass spectrometer (Thermo Fisher Scientific, Waltham, MA, USA) with data-dependent acquisition (DDA) mass spectrometry. The analysis duration was 60 min, with a positive ion mode and full scan range of 350–1800 m/z. Data were analyzed using MaxQuant 2.1.2.0 software.

### 2.7. Animal Experimental Design

Forty-eight male C57BL/6J mice were obtained from Liaoning Changsheng Biotechnology Co., Ltd., (Shenyang, Liaoning, China). The mice had an average body weight of 20 ± 3 g and were 6–8 weeks old (laboratory animal production license SCXK [Liao] 2020-0001). The mice were housed in an environment with automatic regulation of relative humidity ranging from 40% to 70% under a 12 h light/dark cycle at 25 ± 2 °C. All mice were fed standard feed (Jiangsu Synergy Pharmaceutical and Biological Engineering Co., Ltd., Nanjing, Jiangsu, China) and provided with pure water ad libitum during the experiment under pathogen-free conditions in accordance with animal care standards. In the experimental process, a double-blind design was implemented to avoid the influence of subjective biases. The Heilongjiang Bayi Agricultural University’s Ethics Committee of Animal Studies approved the animal studies, which were designed in compliance with the Laboratory Animal Administration Regulations (project identification code: SPXY2024004; date of approval: 29 February 2024).

**Figure 1:**
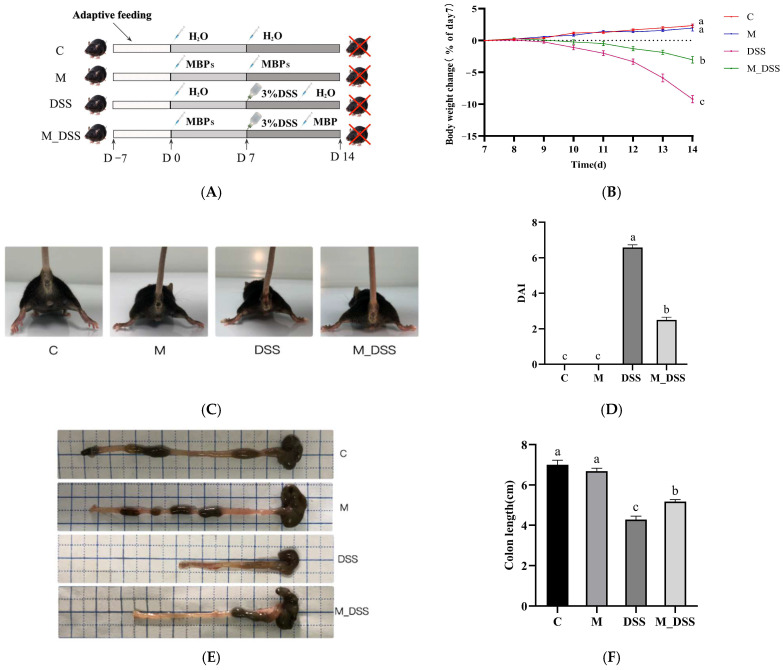
Design of animal experiments and effects of MBPs on colitis symptoms. (**A**) Design of the animal experiments. (**B**) Representative photographs of mice in the four groups after modeling. (**C**) Change in body weight (% on day 7). (**D**) DAI score. (**E**) Representative images of the mouse colon. (**F**) Colon length. Data are presented as mean ± SEM (*n* = 12). a, b, and c denote statistically significant differences between the groups (*p* < 0.05). Groups marked with the same letter do not have significant differences, whereas groups marked with different letters have significant differences.

Following one week of acclimatization, the mice were divided into four groups using a random number generator based on body weight factors: control (C); DSS-induced colitis (DSS); MBPs (M); and MBPs and DSS-induced colitis (M_DSS) (Figure 1A). Throughout the experiment, groups M and M_DSS were administered 200 mg/kg MBPs daily, whereas groups DSS and C were administered an equal amount of pure water. From day 8, the DSS and M_DSS groups were provided with water containing 3% DSS (MP Biomedicals, Santa Ana, CA, USA) ad libitum instead of pure water for 7 days, whereas the other groups continued to receive pure water. On day 15, blood was collected from the eyes of all mice under isoflurane anesthesia (Hebei Yipin Pharmaceutical Co., Ltd., Shijiazhuang, Hebei, China), and the mice were euthanized by dislocation. Colon length was measured, and colon contents were gathered and stored at 80 °C for further examination. Colonic tissues were fixed with 4% paraformaldehyde (*w*/*v*) solution.

### 2.8. Disease Activity Index (DAI)

During the trial, the body weight, stool consistency, and fecal blood of the mice were monitored daily and used as indicators to calculate the DAI. The sum of each item constituted the final DAI score (Supplementary Table S1).

### 2.9. Histological Analysis of the Colon

The preserved colonic tissues were stained with hematoxylin and eosin (H & E), embedded in paraffin, and sliced into 4 μm sections using a microtome for histological examination. Pathological changes in the samples were observed at 200× and 400× magnifications using a light microscope. Histological evaluation was performed using a scoring system (Supplementary Table S2) [31].

### 2.10. Immunohistochemistry Staining

The slides were subjected to antigen retrieval by dewaxing, rehydrating, rinsing with distilled water, and immersion in a citric acid antigen repair solution (pH 6.0). Endogenous peroxidase was inhibited by incubation with 3% H_2_O_2_ at 25 °C. After rinsing three times for 5 min with phosphate-buffered saline (PBS; pH 7.4), the slides were blocked with 10% bovine serum albumin blocking buffer for 30 min. Subsequently, the slides were incubated with primary anti-Claudin-1 and anti-ZO-1 antibodies overnight at 4 °C. After rinsing, the secondary antibodies were added to the samples and incubated for 50 min at room temperature (around 25 °C) in the dark. The slides were rinsed three times for 5 min with PBS and stained with diaminobenzidine (DAB) reagent. Images were acquired using a microscope and analyzed using ImageJ software (version 1.53k) to calculate the DAB staining positive regions.

### 2.11. Measurement of Inflammatory Cytokines in the Serum

Thirty minutes after blood collection from the eyes, the blood was centrifuged at 4 °C and 3500 rpm for ten minutes to separate the serum. The recovered supernatant was used to detect IFN-γ, TNF-α, IL-1β, IL-6, and IL-10 using ELISA kits (Shanghai Beyotime Biotechnology Co., Ltd., Shanghai, China).

### 2.12. Analysis of Gut Microbiota in Colon Content

Bacterial DNA was extracted from the colon contents using a MagPure Soil DNA LQ Kit (Guangzhou Magen Biotechnology Co., Ltd., Guangzhou, Guangdong, China). DNA content and integrity were analyzed using a NanoDrop 2000 spectrophotometer (Thermo Fisher Scientific, Waltham, MA, USA) and agarose gel electrophoresis, respectively. Using two universal primer pairs (343F:5′-TACGGRAGGCAGCAG-3; 798R:5′-AGGGTATCTAATCCT-3), the V3-V4 hypervariable regions of the bacterial 16S rRNA gene were amplified by polymerase chain reaction (PCR) in a 30 μL reaction in a PCR thermocycler (Bio-rad 580BR10905). The PCR products were purified using Agencourt AMPure XP beads (Beckman Coulter Co., Brea, CA, USA) and quantified using the Qubit dsDNA test kit. Subsequently, the concentrations of the products were adjusted for sequencing. The Illumina NovaSeq6000 was used for sequencing, with two paired-end read cycles of 250 bases each (Illumina Inc., San Diego, CA, USA; OE Biotech Company, Shanghai, China).

The raw sequencing data were in FASTQ format. Paired-end reads were preprocessed using the Cutadapt software (2020.11.1) to detect and remove adapters. After trimming, paired-end reads were filtered for low-quality sequences, denoised, merged, and chimera reads were detected and removed using DADA2 with the default parameters of the QIIME2. Finally, the software output the representative reads and an ASV abundance table. The representative reads of each ASV were selected using the QIIME 2 package. All representative reads were annotated and blasted against the Silva database Version 138 using the q2-feature-classifier with default parameters. Sequencing depth was evaluated using diversity rarefaction curves. The microbial diversity in the colon content samples was estimated using alpha diversity metrics (Shannon index). The binary Jaccard distance matrix, generated using QIIME software (2020.11), was used for binary Jaccard Principal coordinates analysis (PCoA) and phylogenetic tree construction. The compare_categories.py tool in QIIME2 was used to perform PERMANOVA/Adonis analysis to assess the significance of group differences. Using the Kruskal–Wallis statistical algorithm, LEfSe was employed to perform a differential abundance analysis of species abundance. 16S rRNA gene amplicon sequencing and analysis were conducted by OE Biotech Co., Ltd. (Shanghai, China).

### 2.13. Statistical Analysis

The data were evaluated using one-way ANOVA and Tukey’s test and are presented as the mean ± standard error of the mean (SEM) using SPSS v.26 software. GraphPad Prism v.8 software was used for image processing. Statistical significance was set at *p* < 0.05.

## 3. Results

### 3.1. Scanning Electron Microscope Analysis

**Figure 2:**
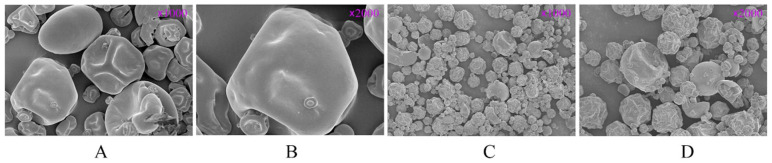
SEM images of mung bean protein and MBPs at 1000× and 2000× magnifications. (**A**,**B**) Mung bean protein; (**C**,**D**) MBPs.

Figure 2 shows the scanning electron microscopy (SEM) images of mung bean protein and MBPs at 1000× and 2000× magnifications. Both mung bean protein and MBPs exhibited clear spherical particles, with a smooth surface. The MBPs obtained after hydrolysis had smaller particle sizes and irregular folds on their surfaces.

### 3.2. FTIR Spectroscopy Analysis

**Figure 3:**
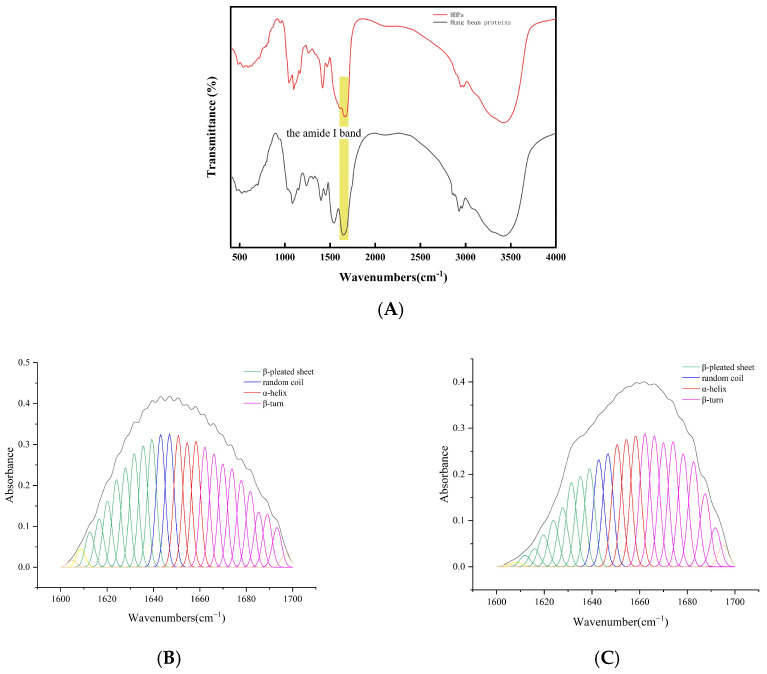
(**A**) FTIR spectra of mung bean protein and MBPs. (**B**,**C**) Amide I band FTIR second derivative curve fitting of mung bean protein and MBPs in the range of 1600~1700 cm^−1^.

FTIR was used to investigate the structural variations in mung bean protein before and after hydrolysis. Secondary structure studies of proteins analyzed using FTIR spectroscopy are mainly based on the amide Ι band. The FTIR spectra and amide I band second derivative curve fitting of both mung bean protein and MBPs are presented in Figure 3. The ratios of the different secondary structures were computed using baseline correction, deconvolution, and second-order derivative fitting of the amide I band within the infrared spectra. This was achieved by referring to the attribution table of the protein secondary structure corresponding to the peak position of the amide I band and the peak area of each subpeak (Table 1). The findings revealed that the secondary structures of mung bean protein and MBPs manifested in diverse forms. In mung bean protein, β-pleated sheets and β-turns are predominant in its secondary structure. However, when papain hydrolyzed mung bean protein into MBPs, a transformation occurred. Specifically, the proportions of α-helix and random coil structures remained relatively stable, whereas a portion of the β-pleated sheet was converted into the β-turn. These results indicate that the original hydrogen bond network was disrupted, leading to a decrease in the content of ordered α-helices and β-sheets. This caused the ordered structure of the protein to become disordered and the molecular structure to become more relaxed. The increase in β-turn content suggests a more flexible secondary structure [32]. This enables them to participate in various biological processes and exhibit higher biological activity. Studies have shown that the specific conformation of β-turns is to some extent dependent on the amino acid sequence. Enzymatic digestion disrupts the original peptide chain structure, exposing more amino acid residues, which, to some extent, forces the formation of β-turns [33].

### 3.3. Amino Acid Composition of MBPs

The amino acid composition of MBPs is presented in Table 2 (g/100 g MBPs). Tryptophan and cysteine were not measured because they were destroyed by acid hydrolysis during the pretreatment. Given that mung bean protein is rich in lysine, which is generally the first limiting amino acid in cereals, the lysine content in MBPs was relatively high, reaching 4.728 g/100 g. This is notably higher than that of other cereal peptides, endowing MBPs with enhanced nutritional values. Additionally, glutamic acid exhibited the highest amino acid content in MBPs, followed by aspartic acid.

### 3.4. Molecular Weight Distribution of MBPs

A mixture of MBPs was obtained after hydrolyzing mung bean protein with papain. According to Table 3, which presents the relative molecular weight distribution of the MBPs, the majority of the MBPs had a molecular weight concentrated within 1000, accounting for 77.12% of the total. MBPs with molecular weights between 1000 and 2000 also constituted a significant portion, at 15.32%, while those with molecular weights exceeding 2000 formed a relatively small fraction, at 7.57%.

### 3.5. MBPs Peptide Sequences

Using LC-MS/MS, 274 peptide sequences corresponding to 40 proteins were identified in mung bean peptides. Using Peptide Ranker to screen for bioactive peptides with a score greater than 0.9, six peptide segments were selected (Table 4). Papain preferentially hydrolyzes peptide bonds at the N-terminus of amino acids with two carboxyl groups and aromatic amino acids; it can also hydrolyze the carboxyl-terminal ends of arginine and lysine. Therefore, the carboxyl-terminal ends of five out of the six peptides meet the active site of papain, and the amino acids exposed at the carboxyl-terminal ends of these five peptides (phenylalanine (F) and leucine) are all hydrophobic, which enhances the hydrophobicity of the mung bean peptides. The carboxyl-terminal end of the remaining peptide is composed of arginine. Studies have shown that arginine (R) is a characteristic amino acid of anti-inflammatory peptides [34].

### 3.6. Effects of MBPs on Colitis Symptoms

Mice in the DSS group exhibited poor physical condition during DSS treatment, characterized by reduced activity, significant weight loss, diarrhea, bloody stools, and pale extremities, indicating successful modeling (Figure 1B,C). In contrast, the symptoms in the M_DSS group were milder than those in the DSS group. Notably, no significant variations in appearance, behavior, or body weight were observed in the C and M groups, suggesting that MBPs have no substantial toxicity or adverse effects.

The DAI, a crucial index for evaluating colitis, assesses enteritis based on three aspects: weight loss, stool properties, and degree of bloody stools. Higher scores indicate more severe disease. At the end of the experiment, the DSS group had the highest DAI score, reflecting the most severe inflammation (Figure 1D). Administration of MBPs prior to DSS induction in the M_DSS group significantly reduced the score by 62% compared to that in the DSS group. However, a significant difference remained between the M_DSS and C-groups. The DAI scores of the M group were identical to those of the C group.

Colon length is a morphological indicator of the degree of colitis [27]. In the DSS group, the colon length was 61% of that in the C group, with a significant difference between the two (Figure 1E,F). In contrast, the colon length in the M_DSS group was significantly higher (by 21%) than that in the DSS group, although it did not reach the level of the C group and remained significantly different. No significant differences in colon length were observed between the M and C groups. These results indicate that MBPs can alleviate DSS-induced colitis in mice.

### 3.7. Effects of MBPs on Colon Tissue Damage

**Figure 4:**
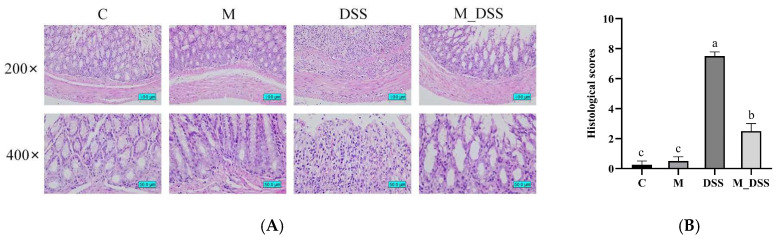
Effect of MBPs on colon tissue damage. (**A**) H & E staining representative photos of colons from mice, at 200× and 400× magnification. (**B**) Histological scores. Data are presented as mean ± SEM (*n* = 4). a, b, and c denote statistically significant differences between groups (*p* < 0.05). Groups marked with the same letter do not have significant differences, while groups marked with different letters have significant differences.

The severity of colonic inflammation was evaluated by pathological assessment of colonic tissues using H&E staining. The results (Figure 4) revealed significant differences in the degree of damage among the different groups of mice. In the M and C groups, the colonic tissues appeared normal, with no abnormal lesions. The crypts exhibited typical morphology, the mucosal layer glands were neatly arranged, and there was no inflammatory cell infiltration. Goblet cells were regularly arranged and abundant. In contrast, the overall structure of colonic tissue in the DSS group was abnormal. Some mucosal epithelial cells were necrotic and sloughed off, the glandular structure of the mucosal layer was largely absent, goblet cells were depleted, nuclei were fixed and lysed, and the tissue was infiltrated by inflammatory cells, indicating that DSS damaged the colonic tissue. Prominent improvements in the morphological changes associated with colitis were observed in the M_DSS group. MBPs alleviated these symptoms compared to those in the DSS group. Overall structural abnormalities in the colonic tissues were reduced, the number of goblet cells increased, the glands in the mucosal layer were more regularly arranged, and the tissues showed less necrosis. Although inflammatory cell infiltration was still present, it was significantly reduced compared to that in the DSS group. The histopathological scores in the DSS group were considerably higher than those in the other groups, indicating the most severe colonic tissue damage following DSS treatment. However, the scores in the M_DSS group were 67% lower than those in the DSS group owing to pretreatment with MBPs, representing a significant difference. A significant difference still persisted between the M_DSS and C groups, and the M and C groups had the lowest scores, suggesting that MBPs may have mitigated the degree of colon injury caused by DSS.

### 3.8. Effects of MBPs on Intestinal Epithelial Barrier Function

**Figure 5:**
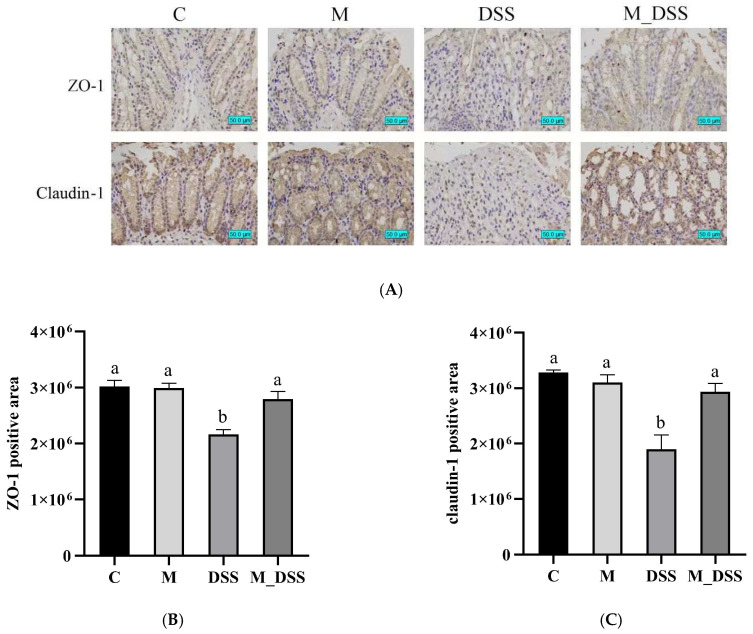
Effect of MBPs on the intestinal epithelial barrier. (**A**) Representative photographs (magnification 400×) of ZO-1 and claudin-1 expression in the colons of mice. (**B**) Graph of the ZO-1 protein-stained area. (**C**) Graph of the claudin-1 protein-stained area. Data are presented as the mean ± SEM (*n* = 4). a and b denote statistically significant differences between groups (*p* < 0.05). Groups marked with the same letter do not have significant differences, while groups marked with different letters have significant differences.

Intestinal epithelial barrier dysfunction is a significant contributor to UC morbidity [35]. TJ proteins play a crucial role in the function of the intestinal epithelial barrier [36]. Claudins are the most important group of TJ proteins and are vital for maintaining permeability [37]. As one of the core TJ proteins, claudins are of utmost importance. ZO-1 is commonly used as an indicator of intestinal barrier function [38]. The colonic tissues of experimental mice were immunohistochemically stained with antibodies against ZO-1and claudin-1. The results (Figure 5) showed that claudin-1 and ZO-1 were abundant and well organized in the colonic epithelial cells in the C group. No significant difference was observed between the M and C groups. In the DSS group, the expression of claudin-1 and ZO-1 in colonic tissue sections was significantly lower than that in the C group, indicating that DSS impaired the colonic epithelial barrier function in mice. In the M_DSS group, the levels of claudin-1and ZO-1 expression and distribution were significantly increased compared to the DSS group and were close to those in the C group, effectively ameliorating the decreased expressions of claudin-1 and ZO-1 in the DSS group. These results suggest that MBPs can effectively restore the expressions of claudin-1 and ZO-1 to enhance intestinal barrier function and mitigate damage to the intestinal epithelial barrier caused by DSS.

### 3.9. Effects of MBPs on Inflammatory Cytokines in Serum

**Figure 6:**
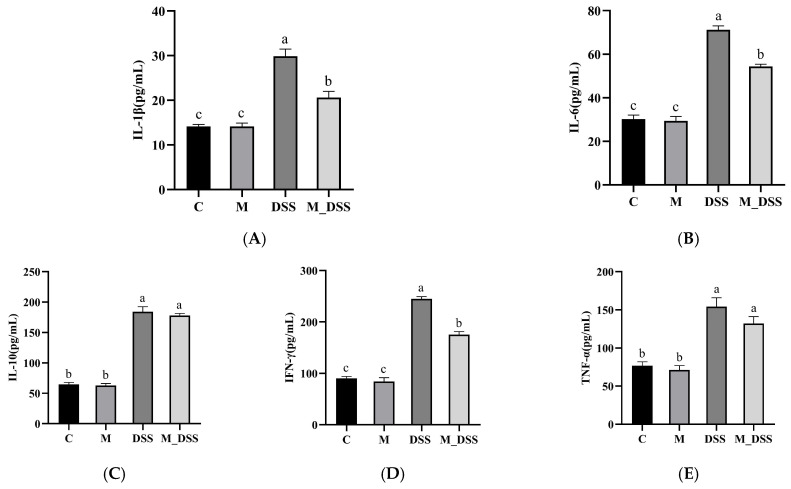
Effect of MBPs on inflammatory cytokines in serum. Data are presented as mean ± SEM (*n* = 6). a, b, and c denote statistically significant differences between groups (*p* < 0.05). Groups marked with the same letter do not have significant differences, while groups marked with different letters have significant differences. (**A**–**E**) represent the levels of inflammatory cytokines IL-1β, IL-6, IL-10, IFN-γ, and TNF-α in the serum respectively.

Inflammatory cytokines are important mediators of colonic inflammation and reflect the degree of colonic injury [39]. Therefore, this study investigated the effect of MBPs on the levels of inflammatory cytokines in the serum of mice with UC. The results (Figure 6) showed no significant difference in the levels of serum anti-inflammatory and pro-inflammatory cytokines between the M and C groups, indicating that MBPs did not affect the inflammatory cytokine levels in normal mice. In contrast, the DSS group exhibited significantly higher levels of inflammatory cytokines (IFN-γ, TNF-α, IL-10, IL-6, and IL-1β) than the C group. Specifically, the levels in the DSS group were 2.72, 2.0, 2.84, 2.36, and 2.1 times higher than those in the C group, respectively, indicating that DSS administration led to a notable increase in inflammatory cytokine levels in the serum of mice. In the M_DSS group, MBPs significantly downregulated the elevated pro-inflammatory cytokine levels (IFN-γ, TNF-α, IL-6, and IL-1β) induced by DSS in the serum of mice. The downregulation ratios were 28%, 14%, 24%, and 30%, respectively. However, the expression of pro-inflammatory cytokines in the M_DSS group remained significantly higher than that in the C group, and there was a significant difference between the two groups. This indicates that MBPs significantly inhibited the overexpression of IFN-γ, IL-6, and IL-1β, although the degree of inhibition of inflammatory cytokines did not reach that of the C group. There were no significant effects of MBPs on IL-10 and TNF-α levels in the serum of DSS-induced colitis mice. This result may be due to the fact that TNF-α is a multifunctional pro-inflammatory cytokine that acts through various signaling pathways, affecting multiple cell types and physiological processes. Although TNF-α plays a central role in inflammatory responses, its mechanism of action is complex and involves multiple signaling pathways and targets. Therefore, active substances can exert a more significant impact on other inflammatory cytokines by directly or indirectly inhibiting these signaling pathways [40].

### 3.10. Effects of MBPs on Gut Microbiota Diversity

**Figure 7:**
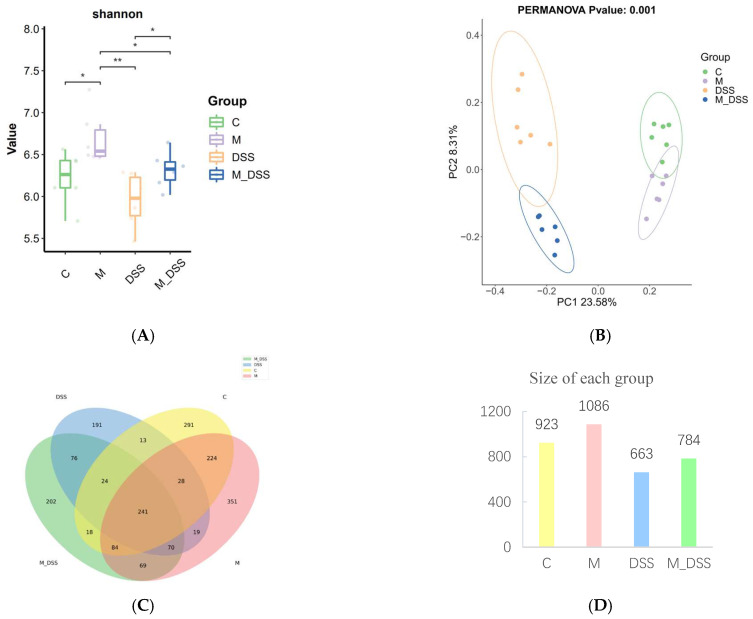
Effect of MBPs on gut microbiota diversity. (**A**) Shannon index. (**B**) PCoA. (**C**) Venn diagram. (**D**) Total number of species in each group. * *p* < 0.05, ** *p* < 0.01.

The diversity rarefaction curve tended to flatten as the number of sequences increased, indicating that the sequencing depth was sufficient to capture the sample diversity. Various alpha diversity indices were calculated to assess species richness and evenness within the samples, providing insights into the overall diversity of the biological community. The α diversity values of the DSS group were lower than those of the other three groups, although the difference was not statistically significant between the DSS and C groups (Figure 7A). The α diversity index of the M_DSS group was higher than that of the DSS group, with a significant difference, and it was slightly higher than or close to that of the C group. The M group exhibited higher alpha diversity indices than all other groups and showed significant differences compared to the C group. These results suggest that MBPs can increase the diversity and uniformity of intestinal microbiota in normal mice but have no significant effect on the diversity and uniformity of intestinal microbiota in mice with UC.

Beta-diversity analysis was used to assess the similarity and variability of communities within different groups. The results (Figure 7B) indicated that the C group was completely distinct from the DSS group, the M_DSS group was differentiated from the DSS group, and the M group was partially differentiated from the C group. These results indicate that MBPs can alter the composition of intestinal microbiota.

Based on the ASV analysis, a Venn diagram was used to compare the richness of species among the groups. The results (Figure 7C,D) showed that the groups had 241 identical species, and the total numbers of species in the C, M, DSS, and M_DSS groups were 923, 1086, 662, and 784, respectively, with 291, 351, 191, and 202 endemic species. Mice with DSS induction had a reduced gut microbiota; however, MBPs enriched the gut microbiota species and increased the number of endemic species in both normal and DSS-induced mice. These results indicate that MBPs can improve the variety and composition of the intestinal microbiota.

### 3.11. Effects of MBPs on the Gut Microbiota Structure

**Figure 8:**
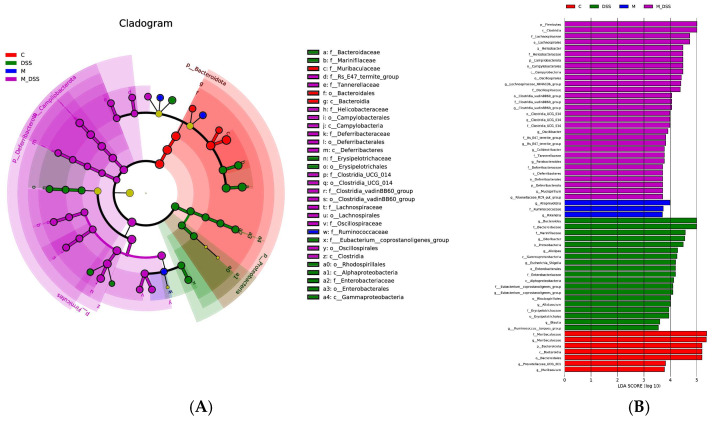
Effect of MBPs on the characteristic differences in the gut microbiota. (**A**) Taxonomic cladogram from LEfSe. (**B**) Differentially abundant taxa’s LDA scores. The names of the communities that could not be shown in the Figure 8A as biomarkers are shown on the right side of the clade diagram (only different communities from phylum to family are displayed). Communities with LDA scores > 3.5.

**Figure 9:**
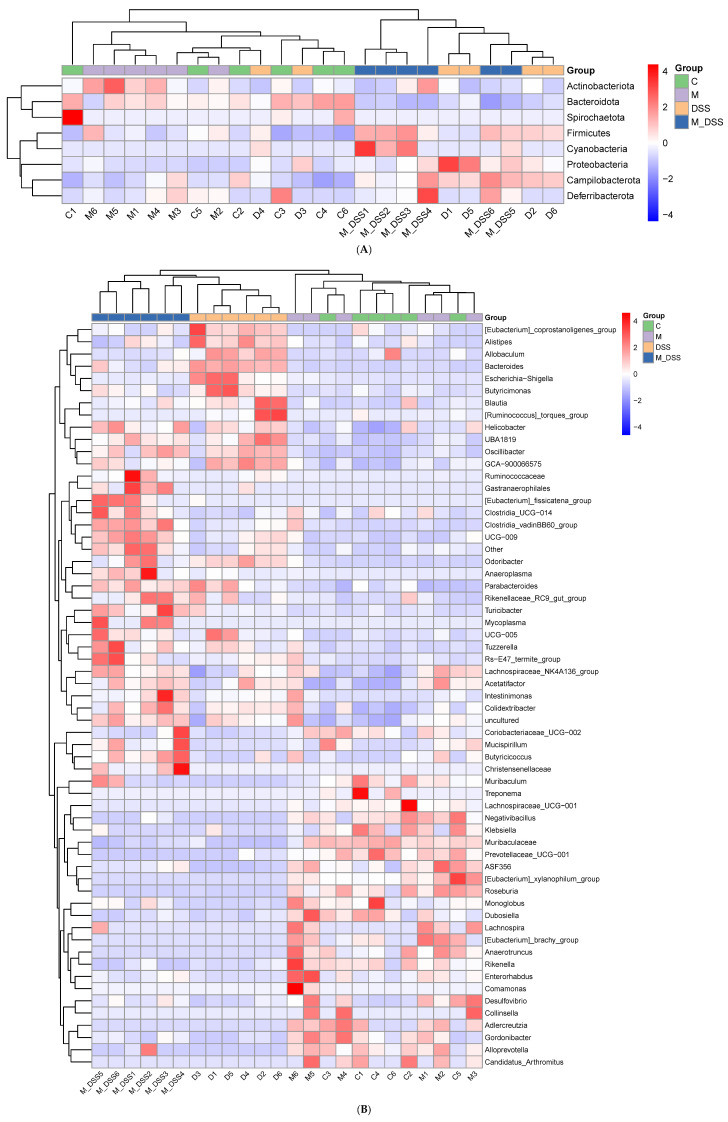
(**A**,**B**) Heatmaps showing the differential species abundance at the phylum and genus levels, respectively. The clustering tree on the left side of the figure represents the species clustering tree; the upper clustering branch group indicates that the samples come from different groups. Red color indicates a higher relative abundance of species, while blue color indicates a lower relative abundance of species.

In the linear discriminant analysis effect size (LEfSe) investigation, emphasis was placed on statistical significance and biological relevance. Figure 8 shows communities with LDA scores > 3.5 as determined by the LEfSe analysis (Kruskal–Wallis, *p* < 0.05). A total of 4, 11, 1, and 20 communities with intergroup differences were detected from the phylum to the family level in the C, DSS, M, and M_DSS groups, respectively. Among them, the significantly different communities observed in the DSS group were Proteobacteria, Bacteroida ceae, Marinifilaceae, Erysipelotrichaceae, Erysipelotrichales, Eubacterium coprostanoligenes, Rhodospirillales, Alphaproteobacteria, Enterobacteriaceae, Enterobacterales, and Gammaproteobacteria. The most significantly different community observed in the M group was Ruminococcaceae. The significantly different communities in Group C were Bacteroidales, Bacteroidia, Bacteroidetes, and Muribaculaceae. These results represent the dominant microbiota in each group after comparison. Simultaneously, statistical analysis was conducted at the phylum and genus levels, and a heatmap was plotted based on the relative abundance of differential species (Figure 9). This allows for a clear visualization of the differences in microbial species and their relative abundances at each level, facilitating a better visualization of microbial changes. Additionally, after processing the relative abundance data of microbial communities from each sample using the min–max normalization method, a stacked bar chart was created using Excel software to analyze the community structure.

**Figure 10:**
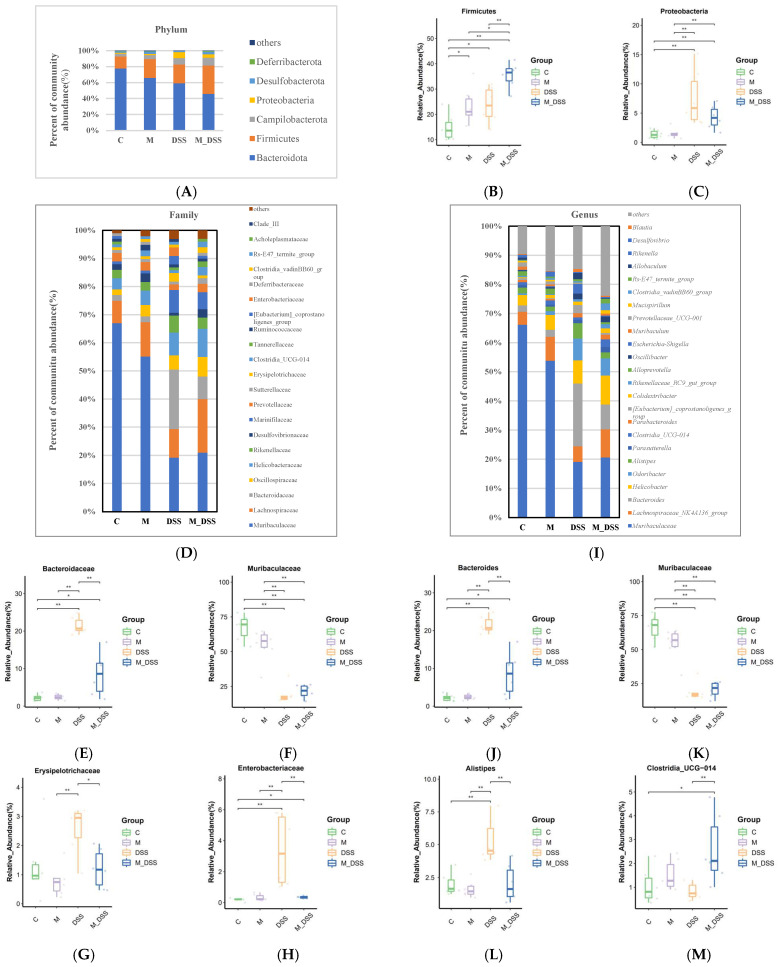
Effect of MBPs on changes in the relative abundance of gut microbiota. (**A**) Phylum level. (**B**) Firmicutes. (**C**) Proteobacteria. (**D**) Family level. (**E**) Bacteroidaceae. (**F**) Muribaculaceae. (**G**) Erysipelotrichaceae. (**H**) Enterobacteriaceae. (**I**) Genus level. (**J**) Bacteroides (**K**) Muribaculaceae. (**L**) Alistipes. (**M**) Clostridia_UCG-014. * *p* < 0.05, ** *p* < 0.01.

At the phylum level (Figure 10A–C), the microbial composition of each group, in order of relative abundance reduction, mainly consisted of six phyla: Bacteroidetes, Firmicutes, Campilobacterota, Proteobacteria, Desulfobacterota, and Deferribacterota. Bacteroidetes and Firmicutes species accounted for over 80% of the gut microbiota. Compared with the C group, DSS treatment changed the abundance of Bacteroidetes, Deferribacterota, and Desulfobacterota and increased the relative abundance of Proteobacteria. In the M_DSS group, MBPs improved the decline in the relative abundance of Deferribacterota and Desulfobacterota and the increase in the relative abundance of Proteobacteria caused by DSS treatment. The relative abundances of Firmicutes and Campilobacterota also changed in the M_DSS group.

At the family level (Figure 10D–H), the intestinal microbiota of the mice mainly consisted of Muribaculaceae, Lachnospiraceae, Bacteroidaceae, Oscillospiraceae, Helicobacteraceae, Rikenellaceae, Desulfovibrionaceae, Marinifilaceae, and others. The abundance of Bacteroidaceae, Rikenellaceae, Marinifilaceae, and Enterobacteriaceae increased, while the abundance of Muribaculaceae significantly decreased in the DSS group compared to the C group. MBP treatment differentially inhibited these changes, just as it reversed the increase in the abundance of Bacteroidaceae and Enterobacteriaceae caused by DSS. In this study, the abundance of Erysipelotrichaceae in the DSS group increased compared to that in the C group, but there was no significant difference between the two groups. MBP treatment significantly inhibited the increase in Erysipelotrichaceae abundance caused by DSS. In addition, Desulfovibrionaceae and Lachnospiraceae were found in greater abundance in the intestinal contents of mice treated with MBPs. At the genus level (Figure 10I–M), the gut microbiota was compared between the groups. The abundances of *Muribaculaceae* and *Muribaculum* decreased substantially, whereas those of *Bacteroides*, *Odoribacter*, *Alistipes*, *[Eubacterium]_coprostanoligenes_group*, *Escherichia–Shigella*, *Allobaculum*, and *Blautia* were significantly higher in the DSS group than in the C group. However, MBP treatment significantly reduced the DSS-induced increase in the abundance of *Bacteroides*, *Alistipes*, *[Eubacterium]_coprostanoligenes_group*, *Escherichia-Shigella*, and *Allobaculum* and increased the relative abundance of *Clostridia _UCG_014*.

### 3.12. Effects of MBPs on Enrichment Analysis of Intestinal Microbiota Pathway

**Figure 11:**
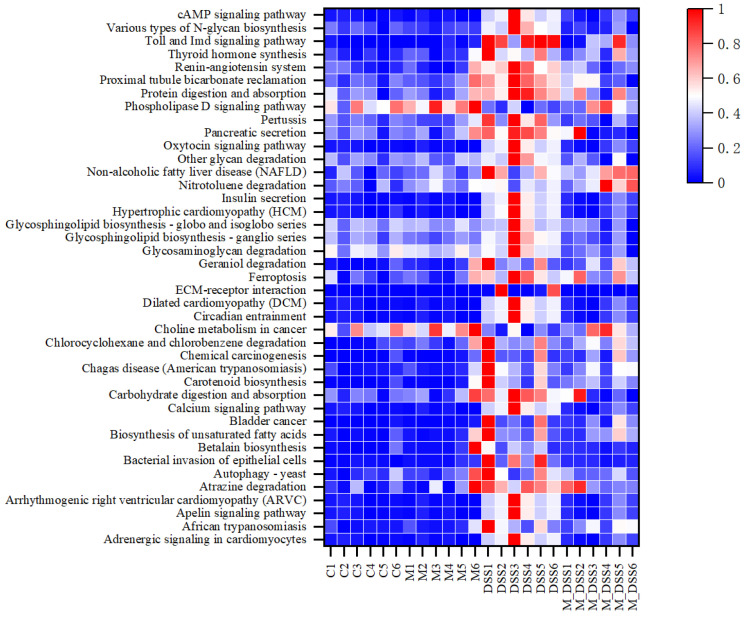
Effect of MBPs on the enrichment of gut microbiota pathways.

In this experiment, based on the analysis of the KEGG database, 263 significantly altered signaling pathways were identified by comparing the functional alterations in the gut microbiota among the groups. A total of 43 signaling pathways were screened, in which MBPs acted and inhibited DSS-induced changes (Figure 11).

MBPs significantly downregulated the Phospholipase D signaling pathway and choline metabolism in the cancer signaling pathway induced by DSS. The remaining 41 signaling pathways were significantly upregulated after DSS treatment. In the M_DSS group, pancreatic and insulin secretion signaling pathways upregulated by MPBs were restored to levels similar to those of the C group. MBPs also inhibited the significant upregulation of unsaturated fatty acid biosynthesis, ferroptosis, protein digestion and absorption, and Toll and Imd signaling pathways in DSS-induced colitis.

### 3.13. Correlation Analysis of Colitis Indicators and Gut Microbiota Under the Influence of MBPs

**Figure 12:**
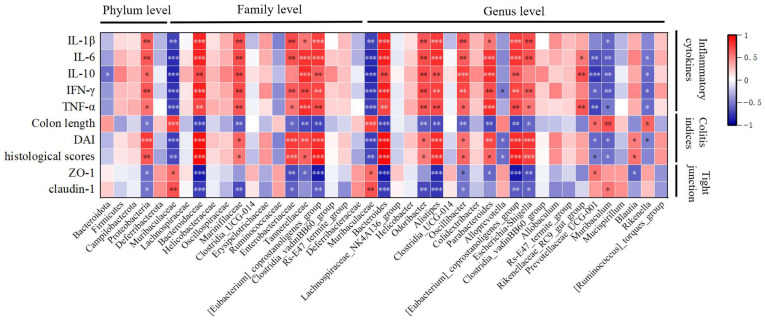
Correlation analysis heat map of colitis indicators and gut microbiota under the influence of MBPs. * *p* < 0.05, ** *p* < 0.01, *** *p* < 0.001.

**Figure 13:**
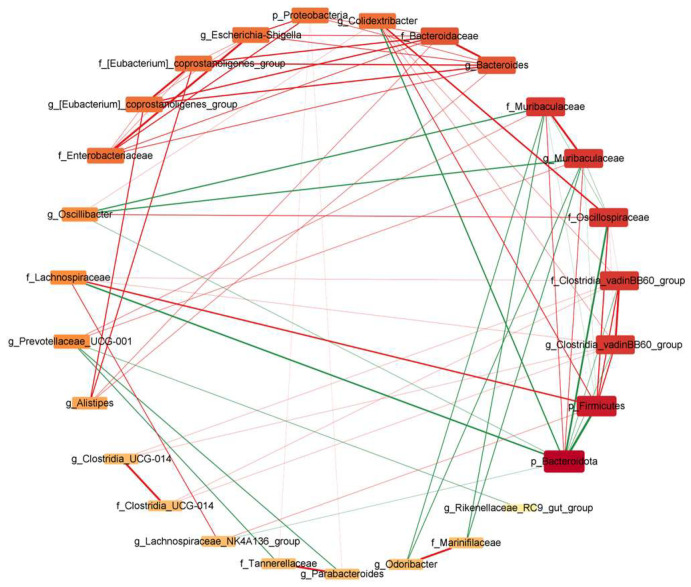
Microbial correlation network analysis under the influence of MBPs. Red lines represent positive correlations, and green lines represent negative correlations. The thickness of the lines represents the magnitude of the correlation coefficients.

The association between gut microbiota and inflammation in UC was studied using Pearson’s correlation coefficients. By creating a correlation heatmap between species significantly different between groups at the phylum, family, and genus levels, as obtained from LEfSe analysis with an LDA score > 3.5, and colitis indicators (including colitis index, inflammatory cytokines, and TJ proteins), it was possible to identify gut microbiota highly correlated with colitis indicators. Since these microbiota undergo significant changes in abundance due to treatment with DSS and MBPs, they can be considered characteristic microbiota of DSS and MBPs. Figure 12 shows that the Muribaculaceae, *Muribaculaceae*, *Prevotellaceae_UCG_001*, *Muribaculum*, and *Rikenella* genera were positively correlated with the TJ proteins and colonic length and inversely correlated with DAI, histological scores, and inflammatory cytokines. These can be considered as beneficial bacteria related to MBPs. The other gut microbiota shown to have significant correlations in the figure exhibited correlations that are opposite to those of the aforementioned microorganisms. These bacteria can be considered harmful and are associated with DSS. Figure 13 is based on Pearson correlation analysis and uses species obtained from LEfSe analysis with a *p*-value < 0.05 and a correlation coefficient greater than 0.7 to draw a correlation network analysis diagram, presenting the results of a correlation network analysis among microorganisms. In the figure, nodes connected to more other nodes indicate higher associations with other microorganisms, thereby allowing us to understand the interaction between microorganisms. The nodes in the figure are arranged clockwise from the smallest to the largest based on the number of edges connected to them. From the figure, it can be seen that Bacteroidota is most closely related to other microorganisms.

## 4. Discussion

UC is a chronic, relapsing inflammatory bowel disease that significantly compromises the quality of life of patients. Although pharmacological interventions are the mainstay of UC management, prolonged use often results in substantial adverse effects. Mung bean-derived peptides (MBPs) are bioactive peptides with intrinsic anti-inflammatory potential; however, their precise efficacy and underlying mechanisms in UC treatment remain insufficiently characterized. This study aimed to elucidate the therapeutic effects and mechanistic pathways of MBPs in UC treatment.

Bioactive peptides are typically short protein fragments comprising 2–20 amino acid residues. Their relatively low molecular weights confer unique structural and functional advantages, including limited spatial folding and enhanced amino acid exposure. These features render the peptides more resistant to proteolytic degradation by endopeptidases, thus preserving their biological activity, particularly in modulation of inflammatory responses [34]. In our study, MBPs were obtained via enzyme-specific hydrolysis of mung bean protein. Structural characterization revealed significant differences in the surface morphologies of the parent mung bean protein and the resulting MBPs. These differences are primarily attributed to the enzymatic cleavage of peptide bonds, which reduces the peptide chain length and leads to the formation of smaller fragments [41]. Notably, over 90% of the peptides in the MBPs contain less than 20 amino acid residues, with approximately 80% comprising fewer than 10 amino acid residues. The high proportion of low-molecular-weight peptides enhances their anti-inflammatory potential [42]. At the same time, this release is accompanied by the destruction of the protein’s spatial structure and the exposure of hydrophobic groups within due to the action of papain, thereby increasing hydrophobicity and its biological activity. Hydrophobic amino acids are commonly found in bioactive peptides with immunomodulatory properties [34,43]. Their presence facilitates membrane interactions, which can modulate cellular signaling cascades and suppress inflammatory responses [43]. For example, Chen et al. demonstrated that alanine significantly enhances peptide hydrophobicity, thereby amplifying its anti-inflammatory activity [44]. In the present study, the hydrophobic amino acid content of MBPs was quantified as 20.646 g/100 g, indicating substantial enrichment that likely contributes to their potent bioactivity. Thus, the unique structural attributes of MBPs, particularly their low molecular weight and hydrophobicity, are closely associated with their therapeutic potential for mitigating UC-associated inflammatory responses.

Disruption of the intestinal epithelial barrier is widely recognized as a critical factor in the pathogenesis of UC. Increased permeability of the mucosal lining allows pathogenic microorganisms to penetrate the lamina propria, eliciting aberrant immune and inflammatory responses [45]. Restoration of epithelial integrity, particularly via the regeneration of goblet cells in the colon, has been shown to attenuate DSS-induced intestinal mucosal damage [11]. In this study, the administration of MBPs alleviated hallmark UC symptoms in DSS-induced mice, including diarrhea, hematochezia, bodyweight loss, and colonic shortening. Notably, MBPs promoted the recovery of goblet cell numbers and restored the expression levels of TJ proteins, such as claudin-1 and ZO-1, both of which were significantly depleted after DSS exposure. These findings suggest that MBPs exert protective effects on colonic tissue by reinforcing the epithelial barrier through the upregulation of TJ proteins and goblet cell regeneration.

DSS treatment is known to increase pro-inflammatory cytokines, including TNF-α, IL-1β, and IL-6, while concurrently suppressing the expression of anti-inflammatory cytokines. IL-10 plays a pivotal role in suppressing inflammatory responses by downregulating pro-inflammatory mediator expression. Interestingly, elevated IL-10 levels observed in the DSS group may reflect a compensatory host defense mechanism [12]. The upregulation of claudin-1 and ZO-1 in the colonic tissues of the MBPs-treated (M_DSS) group may thus be partially attributable to the suppressive effects of MBPs on systemic pro-inflammatory cytokine expression. IL-1β, TNF-α, and IL-6 contribute to TJ protein degradation and compromise barrier integrity [46]. MBP-mediated cytokine modulation likely reduces epithelial permeability, enhances mucosal defense, and mitigates DSS-induced inflammation in the colon.

At the phylum level, gut microbiota analysis revealed that MBP treatment altered the microbial composition in a manner consistent with previously reported results. Specifically, MBPs decreased the relative abundance of Bacteroidetes and increased the proportion of Firmicutes in the gut. This microbial shift parallels the findings of Han et al. [47], who identified an antioxidant peptide from tuna roe that significantly reduced the abundance of Bacteroidetes and Proteobacteria while enhancing Firmicutes abundance, thereby modulating the dominant gut microbiota. Moreover, peptides derived from tuna muscle exhibited sex-specific microbiota effects: they reduced Bacteroidetes and increased Firmicutes in male mice but had the opposite effect in females [48]. The selective enrichment of Firmicutes by MBPs is particularly noteworthy, given the established correlation between Firmicutes abundance and elevated IL-10 levels, which suggests enhanced anti-inflammatory activity [49,50]. In this study, the increased abundance of Firmicutes in MBP-treated mice was positively correlated with IL-10 levels, indicating that MBPs may promote anti-inflammatory effects by favorably reshaping the gut microbial composition. Furthermore, MBP supplementation suppressed DSS-induced expansion of Proteobacteria, a phylum that encompasses numerous opportunistic pathogens. A sustained increase in Proteobacteria, which is rarely abundant in healthy gut microbiota, is often indicative of microbial dysbiosis and has been linked to an increased risk of disease and malnutrition [51]. Thus, the ability of MBPs to prevent Proteobacteria overgrowth reinforces their potential to maintain microbiota equilibrium and mitigate inflammation-related disease.

At the family level, DSS treatment significantly elevated the abundance of Bacteroidaceae, Rikenellaceae, and Marinifilaceae, consistent with previous studies [12,52]. These bacterial families, all members of the Bacteroidetes phylum, play multifaceted roles in gut inflammation [53]. Despite the observed increase in specific families, the overall proportion of Bacteroidetes decreased relative to other phyla, indicating a substantial compositional shift in the gut microbiota after DSS exposure. Importantly, MBP administration appeared to mitigate dysbiosis by suppressing the overgrowth of inflammation-associated taxa, suggesting a stabilizing effect on the gut microbial community. Among the altered families, a notable reduction in the abundance of Muribaculaceae was observed in DSS-treated mice [54]. This decrease is clinically relevant because Muribaculaceae has been associated with immunomodulatory function. Specifically, it contributes to the production of propionate, a short-chain fatty acid that suppresses CD8^+^ T cell activation and promotes immune tolerance [55]. Therefore, its reduction may contribute to an increase in the severity of colitis. In contrast, Erysipelotrichaceae, which is positively correlated with colitis progression [56], showed increased abundance following DSS treatment, which is another indicator of intestinal inflammation. A pronounced increase in Enterobacteriaceae was also noted in the DSS group, consistent with the findings of multiple studies [31,57,58,59]. As a dominant pro-inflammatory bacterial family, Enterobacteriaceae exacerbates colitis by stimulating lipopolysaccharide (LPS) production, which activates innate immune responses and promotes the release of pro-inflammatory cytokines [60]. In our study, MBP treatment significantly suppressed DSS-induced proliferation of Enterobacteriaceae, restoring their levels to those comparable to those in the control group. This was accompanied by a marked reduction in systemic pro-inflammatory cytokine levels, reinforcing the association between microbial modulation and immunological improvements. Correlation analyses further confirmed a strong positive association between Enterobacteriaceae abundance and pro-inflammatory cytokine levels. Interestingly, an increased abundance of Desulfovibrionaceae has been reported to be associated with reduced colitis severity, longer colon length, and elevated IL-10 levels [61,62,63], which are markers of anti-inflammatory and healing responses. In our study, MBP treatment enhanced the relative abundance of Desulfovibrionaceae, supporting its role as a protective species [52]. Similarly, Lachnospiraceae are associated with increased anti-inflammatory cytokine production [31]. Lachnospiraceae are crucial for colitis prevention, and their absence may hasten the development of inflammation [64]. Furthermore, Lachnospiraceae are probiotics that produce short-chain fatty acids, such as butyric acid [46,59]. These findings suggest that MBPs not only suppress pathogenic and pro-inflammatory bacterial families but also promote the enrichment of beneficial anti-inflammatory taxa in the gut. This dual modulation likely contributes to the restoration of microbial homeostasis and alleviation of the symptoms of colitis.

At the genus level, MBP treatment reversed DSS-induced increases in several key pro-inflammatory genera, including Bacteroides, Alistipes, and Escherichia–Shigella. Elevated Bacteroides levels are positively associated with increased disease severity, macrophage infiltration (CD68^+^), and higher levels of IL–1β and TNF–α [52]. The correlation between Bacteroides and inflammatory cytokine levels observed in the present study is consistent with these previous findings. Alistipes, identified as a potential enterotoxin-producing genus, has been frequently reported in patients with IBD [12]. Similarly, Escherichia–Shigella, a common genus within the phylum Proteobacteria, includes pathogenic strains that exacerbate mucosal inflammation and disrupt intestinal homeostasis [65]. Both Allobaculum and Escherichia–Shigella have been recognized as key contributors to DSS-induced microbiota imbalance [66]. In summary, MBPs modulated the gut microbiota composition by decreasing the relative abundance of pro-inflammatory and toxin-producing genera and promoting the proliferation of anti-inflammatory and short-chain fatty acid-producing bacteria. This shift in the microbial profile was closely associated with reduced cytokine-mediated inflammation, restoration of intestinal barrier function, and symptom relief in mice with UC. These findings underscore the therapeutic potential of MBPs in rebalancing gut microbiota and mitigating colitis via a microbiota-mediated mechanism.

The signaling pathways identified through enrichment analysis provide valuable insights into the metabolic and immunological mechanisms by which MBPs exert therapeutic effects in mice with DSS-induced colitis. Among these, the phospholipase D signaling pathway plays a critical role in the viability, proliferation, and metastatic potential of colon cancer cells [67]. In parallel, the choline metabolism pathway is also noteworthy. Choline is a vital source of methyl groups in plants. Perturbations in the gut microbiota enable certain bacterial taxa to metabolize choline, thereby competing with the host for this essential nutrient. This microbial competition can impair choline absorption, significantly affecting the plasma and hepatic levels of methyl donor metabolites [68]. Furthermore, DNA methylation disruptions have been implicated in a wide spectrum of disorders, including cancer, cognitive decline, atherosclerosis, and autoimmune diseases [69,70,71,72]. Another significantly altered pathway was ferroptosis, an iron-dependent form of regulated cell death triggered by excessive lipid peroxidation, which involves the incorporation of polyunsaturated fatty acids into cell membranes. It is closely related to numerous biological activities, particularly those involving amino acids, Fe, and polyunsaturated fatty acids [73]. These findings suggest that upregulated genes in colorectal cancer are associated with the signaling pathways of protein digestion and absorption [74]. In the current study, MBP treatment effectively attenuated the upregulation of the signaling pathways involved in protein digestion and absorption in DSS-induced colitis. Furthermore, Toll and Imd signaling pathways were significantly upregulated in the DSS group, and it has been found that overactive immune responses, particularly immune dysfunction caused by Toll-like receptor (TLR) activation and gut microbiota perturbation, drives the pathogenesis of UC [75]. Overall, these results suggest that MBPs may alleviate the symptoms of DSS-induced colitis by modulating immune-related pathways.

Crucially, our correlation analysis between the intestinal microbiota and UC-related inflammatory markers revealed a strong association between microbial alterations, cytokine levels, disease severity, and the expression of TJ proteins. These findings support the hypothesis that MBPs alleviate colitis by reshaping gut microbial communities and modulating immune and metabolic pathways associated with epithelial barrier function and inflammation.

However, this study exclusively used a DSS-induced UC mouse model. Although this model simulates key aspects of human UC pathophysiology, it may not fully capture the complexity observed in other species or genetic backgrounds. Different animal models may respond differently to MBPs; therefore, extrapolation of the results of this study may be limited. Future research should incorporate multiple animal models for validation to improve the universality and reliability of the results. Second, the current study focused solely on short-term observations. The long-term efficacy and safety of MBPs remain unknown. Prolonged use may elicit unintended systemic effects, and potential adverse reactions remain unknown. Therefore, future research should prioritize long-term follow-up studies to comprehensively assess the sustained therapeutic value and safety profiles of MBPs in chronic diseases.

## 5. Conclusions

We demonstrated that MBPs exert a preventive effect on DSS-induced UC, as evidenced by improvements in DSS-induced weight loss, shortened colonic length, colonic histopathology, impaired gut microbiota, and intestinal barrier dysfunction. In addition, we found that MBPs can suppress inflammation by increasing the expression of TJ proteins, regulating the levels of inflammatory cytokines, decreasing the abundance of harmful bacteria, and increasing the abundance of beneficial bacteria, thereby regulating the expression of immune-related signaling pathways. In summary, this study provides strong support for the use of MBPs as nutritional supplements to prevent UC.

## Figures and Tables

**Table 1:** The attribution table of the secondary structure of proteins corresponding to the peak position of the amide I band and the ratio of each secondary structure in MBPs and mung bean protein.

Scheme 1	α-Helix	β-Pleated Sheet	Β-Turn	Random Coil
the peak position of the amide I band (cm^−1^)	1650–1658	1610–1640	1660–1695	1640–1650
mung bean protein (%)	18.25	33.43	35.65	12.67
MBPs (%)	20.22	23.35	44.73	11.70

**Table 2:** The amino acid composition of MBPs.

Amino Acid Name	Amino Acid Content (g/100g)	Amino Acid Name	Amino Acid Content (g/100g)
Asp	8.568 ± 0.324	Ile	2.513 ± 0.103
Thr	2.263 ± 0.096	Leu	5.131 ± 0.231
Ser	4.142 ± 0.188	Tyr	2.288 ± 0.087
Glu	13.644 ± 0.599	Phe	3.995 ± 0.201
Gly	2.832 ± 0.133	Lys	4.728 ± 0.223
Ala	2.711 ± 0.141	His	1.632 ± 0.0735
Val	3.323 ± 0.158	Arg	4.826 ± 0.256
Pro	2.973 ± 0.132		

**Table 3:** Relative molecular mass distribution of MBPs.

Relative Molecular Mass	Retention Time (min)	Molecular Weight at Peak	Peak Area PERCENTAGE (%)
>10,000	11.566 ± 0.043	16.239	1.66 ± 0.08
10,000~5000	13.677 ± 0.034	5001	0.92 ± 0.04
5000~3000	14.592 ± 0.026	3001	1.80 ± 0.07
3000~2000	15.320 ± 0.044	1999	3.15 ± 0.11
2000~1000	16.292 ± 0.039	1162	15.32 ± 0.66
1000~500	17.437 ± 0.028	614	30.68 ± 1.23
500~180	18.833 ± 0.033	282	35.72 ± 1.50
<180	20.517 ± 0.040	110	10.72 ± 0.06

**Table 4:** Selected peptides derived from MBPs had a Peptide Ranker score of 0.90.

Sequences	Peptide Ranker Score	Molecular Weight	Protein IDs
FPGAF	0.98	537	A0A1S3U4I8
NFFAF	0.98	644	A0A3P9QP39
NPFYF	0.98	686	A0A3P9QP39
SDRWF	0.95	709	A0A3P9QP39
GGGFR	0.93	492	A0A1S3UBT1
NPHRFQDFFL	0.91	1320	A0A3P9QP39

## Data Availability

The original contributions presented in the study are included in the article/Supplementary Materials, further inquiries can be directed to the corresponding author.

## Supplementary Materials

The following supporting information can be downloaded at: https://www.mdpi.com/article/10.3390/foods14081363/s1, Table S1: Disease Activity Index (DAI) scoring system; Table S2: Histological scoring system.
