# Supplementary material for: Mung Bean Peptides Alleviate Dextran-Sulfate-Sodium-Induced Colitis Symptoms in Mice by Protecting the Intestinal Mechanical Barrier and Regulating Gut Microbiota
Source: Foods. 2025 Apr 15;14(8):1363. doi: 10.3390/foods14081363 (PMC12027464; doi:10.3390/foods14081363)
Supplement: Supplementary file 1 [file foods-14-01363-s001.zip › foods-3475935-supplementary.pdf]

Supplementary Table S1 Disease Activity Index (DAI) scoring system

Project / Score	Fecal blood	Stool consistency	Body weight loss
0	No blood	Regular	No
1	Small amount	Soft but formed	1–5%
2	Common blood	Very soft	5–10%
3	Blood in all stools	Diarrhea	10–20%

Supplementary Table S2 Histological scoring system

Project / Score	Inflammation	Depth of inflammation	Crypt damage
0	None	None	None
1	Mild	Mucosa	1/3 damaged
2	Moderate	Submucosa and muscularis	1/2 damaged
3	Severe	Transmural	Only surface epithelium present
4	——	——	Crypts and surface epithelium lost

Note: "——" indicates that the score does not exist.
